# Identification and validation of the role of ZNF281 in 5-fluorouracil chemotherapy of gastric cancer

**DOI:** 10.1007/s00432-024-05838-8

**Published:** 2024-06-16

**Authors:** Yifan Li, Chengying Zhou, Guoxu Wang, Huiru Xin, Yafei Xiao, Changjiang Qin

**Affiliations:** 1 Department of Gastrointestinal Surgery, Huaihe Hospital of Henan University, Kaifeng, Henan Province China; 2 Key Laboratory of Inflammatory Diseases and Immunoregulation, Henan Provincial Health Commission, Kaifeng, China

**Keywords:** Gastric cancer, 5-fluorouracil, ZNF281, DNA repair, Wnt/β-catenin pathway

## Abstract

**Background:**

The early diagnosis of gastric cancer (GC) and overcoming chemotherapy resistance is challenging. The aberrant expression of zinc finger protein 281 (ZNF281) and the over-activation of the Wnt/β-catenin pathway are oncogenic factors and confer tumor chemoresistance. ZNF281 modulates the Wnt/β-catenin pathway to influence malignant tumor behavior. However, the role of ZNF281 in GC chemotherapy and the relationship with the Wnt/β-catenin pathway have not been elucidated by researchers.

**Methods:**

We explored differences in ZNF281 expression in Pan-cancer and normal tissues, the effect of its expression on prognosis of patients treated with 5-fluorouracil (5-FU). Cox regression was utilized to determine whether ZNF281 is an independent prognostic factor. Enrichment analysis was performed to explore the mechanism underlying ZNF281’s role in 5-FU treatment. We assessed the relationship between ZNF281 and the tumour microenvironment (TME) and combined bulk-RNA and single-cell RNA data to analyse the relationship between ZNF281 and immune infiltration. In vitro experiments verified the effects of ZNF281 knockdown on proliferation, invasion, migration, apoptosis, DNA damage of GC cells with 5-FU treated and the Wnt/β-catenin pathway proteins.

**Results:**

ZNF281 was highly expressed in seven cancers and correlates with the prognosis. It is an independent prognostic factor in 5-FU treatment. ZNF281 correlates with TME score, CD8T cell abundance. ZNF281 is primarily associated with DNA repair and the Wnt/β-catenin pathway. ZNF281 knockdown enhanced the effect of 5-FU on phenotypes of GC cells.

**Conclusion:**

We identified and verified ZNF281 as one of the potential influencing factors of 5-FU treatment in GC and may be associated with the Wnt/β-catenin pathway. Low ZNF281 may contribute to improved 5-FU sensitivity in GC patients.

**Supplementary Information:**

The online version contains supplementary material available at 10.1007/s00432-024-05838-8.

## Introduction

Gastric cancer (GC) is a common digestive tract tumor with an increasing incidence worldwide. GC has been recognized as a serious threat to overall health globally. According to Cancer statistics, there were 1,089,103 new cases of GC and 768,793 deaths in 2020 (Sung, Ferlay et al. 2021). Patients with early GC demonstrate better prognosis, with a more than 90% survival rate within 5 years. However, patients with advanced or progressive GC report a less than 30% survival rate within 5 years (Thrift and El-Serag 2020; Zheng, Chen et al. 2020). However, early GC lacks typical clinical symptoms; therefore, it is mostly diagnosed at an advanced stage. Delayed diagnosis limits the choice of treatment modalities. Therefore, the identification of molecular biomarkers is beneficial for early GC diagnosis (Bray et al. 2018).

GC treatment principally involves surgery, chemotherapy, and immunotherapy. Chemotherapy can not only reduce the tumor before surgery but also prolong the survival of the patients (Mittendorf, Jeruss et al. 2011, Becerra, Aquina et al. 2017). Mo et al. found that tumour immune infiltration correlated with response to chemotherapy, and a scoring model based on immune infiltration showed that a high risk score implied stronger DNA repair (Mo, Huang et al. 2020). 5-Fluorouracil (5-FU) is the first-line drug in chemotherapy for GC. It can inhibit the proliferation, migration, and invasive ability of cancer cells by promoting DNA damage apoptosis (Lei, Dong et al. 2022, Wu, Fan et al. 2022). However, some patients develop tolerance during treatment, resulting in low survival rates. Currently, excessive DNA repair was iindetified as one of the mechanisms underlying 5-FU tolerance (Wu, Li et al. 2020). More and detailed mechanisms of GC chemotherapy tolerance and DNA repair deserve to be explored as could provide potential targets for therapeutic sensitization.

The Wnt/β-catenin pathway, also termed the classical Wnt signaling pathway, is central to biogenetic stability, cell proliferation, differentiation, and apoptosis. Wnt/β-catenin dysregulation leads to tumorigenesis, progression, excessive DNA repair, and drug resistance (Klaus and Birchmeier 2008; Koushyar et al. 2020; Caspi et al. 2021; Huang, Sheng et al. 2021). It has been shown that 5-FU can inhibit the malignant biological behaviour of tumours through the Wnt/β-catenin pathway (Zhou, Hu et al. 2020). The activation of abnormal Wnt/β-catenin pathway is one of the mechanisms underlying drug tolerance in GC (Cheng et al. 2018; Hou et al. 2020; Kim, Bae et al. 2023).

The zinc finger protein family is the largest in the human body. it is central to the regulation of cell proliferation, differentiation, and apoptosis (Klug 2010; Sen, Boxer et al. 2012). However, the high expression of its family members leads to the development of several diseases, including tumors (Yang, Hamilton et al. 2008, Yang et al. 2008; Yang, Wang et al. 2008). Zinc finger protein 281 (ZNF281) is a focus of research and a potential target for tumours. Low expression of ZNF281 can inhibit non-homologous repair to promote DNA damage in case of broken DNA structure. Thus, low expression of ZNF281 expression is one of the inhibitors of DNA damage repair. In breast cancer, ZNF281 regulates X-Ray Repair Cross Complementing 2 to affect DNA damage repair and participate in cytotoxic stress response (Pieraccioli, Nicolai et al. 2016). In hepatocellular liver cancer, ZNF281 affects mitochondrial biogenesis through the nuclear respiratory factor 1/peroxisome proliferator-activated receptor gamma co-activator-transcription factor A, mitochondrial axis, triggering cancer progression (Zhao, Zhang et al. 2023). It has been revealed that ZNF281 involved in regulating immune infiltration in cervical and pancreatic cancers(Hou et al. 2022). Additionally, low expression of ZNF281 inhibits tumor progression in oral, breast, and cervical cancers (Ji et al. 2020; Hou et al. 2021; Starzynska et al. 2021; Zeng, Hou et al. 2022). However, the role of ZNF281 in GC chemotherapy and its underlying mechanism have not been explored. Identifying the role of ZNF281 in GC chemotherapy could provide a theoretical basis for theoretical basis for more effective treatment options.

## Materials and methods

### Data acquisition

Expression information of ZNF281 in pan-cancer was obtained from the Tumor Immune Estimation Resource database (TIMER; https://cistrome.shinyapps.io/timer/) and survival information from the Kaplan-Meier plot database (KM; https://kmplot.com/analysis/) for pan-cancer survival information. The mRNA expression data and corresponding clinical information of patients with 5-FU treatment were obtained from The Cancer Genome Atlas Program database (TCGA; https://portal.gdc.cancer.gov/) (Normal = 32; GC = 375) (5-FU cohort = 100; Supplementary Table 1) and KM database (*n* = 34) .GSE13911 (Normal = 38;GC = 31) (Supplementary Table 2) were obtained from Gene Expression Omnibus database (GEO; https://www.ncbi.nlm.nih.gov/geo/).

### Differential expression analysis

Differential expression analyses of ZNF281 were demonstrated with TIMER database and “limma” R package to show its oncogenic effects and diagnostic value. (Ritchie, Phipson et al. 2015). Subsequently, the diagnostic receiver operating characteristic (ROC) curve was plotted for ZNF281. Validation of the differential expression of ZNF281 in AGS, HGC-27, MKN-45 (GC cells) and GES-1(normal gastric epithelial cell) using Western blot.

### Association between ZNF281 and survival

To identify the role of ZNF281 in prognosis for cancers and treatment, survival analyses of ZNF281 in Pan-cancer and 5-FU treatment were performed using the KM database (GSE15459) and “survminer” R package.

### Enrichment analysis

The co-expressed ZNF281 genes in 5-FU cohort were identified using R, and correlation coefficients were identified using “Spearman’s rank correlation.” To explore the mechanism underlying the role of ZNF281 in 5-FU treatment, Gene Ontology (GO) were performed on the positively correlated ZNF281 genes (cor > 0.3; *p* < 0.05) using the David database (https://david.ncifcrf.gov/summary.jsp).

### ZNF281 as an independent prognostic factor

Univariate and multivariate Cox regression analyses were performed to determine whether ZNF281 is an independent prognostic factor for 5-FU cohort.

### Relationship between ZNF281 and the tumour microenvironment (TME)

The “estimate” package was used to score the 5-FU cohort samples: stromal score, immune score, and ESTIMATE score. The samples were divided into high and low ZNF281 expression groups based on the median ZNF281 expression and the “limma” package was used to analyze the difference in scores between the high and low ZNF281 groups. The TIMER database was used to estimate the abundance of immune cells for the 5-FU cohort, and the correlation between ZNF281 expression and immune cell abundance was then analysed (Spearman). To further explore the relationship between ZNF281 and immune infiltration, differences in ZNF281 expression between different cells were analysed using the GSE134520 and GSE167297 single cell RNA (scRNA) datasets from the Tumor Immune Single-cell Hub 2 (TISCH2; http://tisch.comp-genomics.org/home/) database.

### Reagents used in this study

The following reagents were used for the study: ZNF281 rabbit primary antibody (Abcam; USA), c-Myc rabbit primary antibody (Proteintech; China), β-catenin rabbit primary antibody (Proteintech; China), bicinchoninic acid (BCA) kit (Abbkine; China), Wnt/β-catenin pathway protein antibody (Cell Signaling Technology; USA), rabbit secondary antibody (Servicebio; China), enhanced chemiluminescence (Servicebio; China), siRNA (Riobio; China), Lipoo3000 (Thermo Fisher Scientific; USA), Lipoo3000 (Thermo Fisher Scientific; USA), cell lysate (Solarbio; USA), fetal bovine serum (FBS) (Gibico; USA), RPMI-1640 medium (Servicebio; China), trypsin (Servicebio; China), sodium dodecyl-sulfate polyacrylamide gel electrophoresis (SDS-PAGE) (Servicebio; China), 5-FU (Sigma-Aldrich; Germany), dimethyl sulfoxide (DMSO) (Servicebio; China), CCK-8 kit (Abbkine; China), Transwell (8 μm; Corning; USA), matrigel (Corning; USA), crystal violet (Servicebio; China), DNA damage kit (Beyotime Biotechnology; China), apoptosis kit (Abbkine; China).

### Western blot

The cells were collected and mixed with radioimmunoprecipitation assay buffer lysate, and protein concentration was assessed using the BCA protein concentration assay kit. Different protein samples were separated by SDS-PAGE and transferred onto 0.4 μm polyvinylidene fluoride membranes. Subsequently, they were incubated with primary antibody (1:2,000) overnight at 4 °C and with rabbit secondary antibody (1:5,000) for 1 h at room temperature. Protein blot bands were visualized using enhanced chemiluminescence and analyzed using Image J software.

### Cell culture

GC cells, namely, AGS, HGC-27, and MKN-45, and gastric epithelial cells, namely GES-1, were cultured using RPMI-1640 consisting of 10% FBS. They were incubated in 5% CO_2_ at 37 ℃. The cells were passaged at a cell density of approximately 70%. All cells used in this study were obtained from Rosetta Stone Biotech (Taiyuan, China).

### Relationship between ZNF281 and Wnt/β-catenin pathway

In GC, the relationship between ZNF281 and the Wnt/β-catenin pathway requires further explanation. Cellular Myc (c-Myc) and β-catenin were the marker proteins for the Wnt/β-catenin pathway. Differences in cMYC and β-catenin expression between NC and siRNA groups were validated using western blot (Wang et al. 2023). Western blot was performed to detect c-Myc and β-catenin protein expression in the NC and low ZNF281 expression group.(Song, Guo et al. 2021). cMYC and β-catenin rabbit primary antibodies were diluted at a ratio of 1:5,000.

### Cell transfection

ZNF281 was transfected in GC cells using lipoo3000 and siRNA according to the manufacturer’s instructions. The cells were cultured after 96 h, and the transfection efficiency was verified by western blot. Cells after 24 h of culture were ready for functional experiments.

### Detecting cell viability

First, 5-FU powder was configured to a concentration of 5,000 ug/ml using DMSO. Second, the solutions were configured to 0 µg/ml, 5 µg/ml, 10 µg/ml, 25 µg/ml, 50 µg/ml, and 100 µg/ml concentrations. Third, the cells were inoculated at 5,000 cells per well in 96-well plates with varying 5-FU concentrations using RPMI-1640. After 24 h of incubation, 10 µL of Cell Counting Kit-8 (CCK-8) reagent was added to each well and allowed to stand for 0.5 h at 37 °C. The OD value at 450 nm was measured using a spectrophotometer (Biotek; USA). Finally, cell survival at different concentrations was detected as IC50 values using the IC50 Calculator tool (https://www.aatbio.com/tools/ic50-calculator). Subsequent experiments were conducted using the IC50 concentration of 5-FU as a reference. Cellular function experiments were performed in three groups: NC group, 5-FU group, and siRNA + 5-FU group. Cells were seeded at a density of 3,000 cells per well in 96-well plates and incubated for 24 h before being treated with the respective compounds in each group. The OD values at 450 nm were measured at 24, 48, 72 and 96 h as described above.

### Cell migration

Cell counting was performed, and the cells were inoculated onto six-well petri dishes. The bottom surface of the well plates was scratched using a sterile pipette tip. Photographs were captured at 0 h and 24 h, and the results were analyzed using ImageJ. Upon reaching a cell density of 80% or higher, employ a sterile pipette tip to gently scrape the bottom of the plate.

### Transwell cell invasion assay

To begin with, 70 µl of 10% Matrigel was added to the upper chamber and placed in a cell culture incubator for curing. In addition, 200 µl of RPMI-1640 without FBS was added to the transwells and incubated for 30 min at 37 °C. At last, 1 × 105 cells/ml suspension of 200 µl FBS-free RPMI-1640 was added to the upper layer of the Transwell. Subsequently, 800 µl of RPMI-1640 consisting of 10% FBS was added to the lower layer. The plates were incubated for 48 h. Finally, cells in the upper layer were removed, fixed with methanol for 10 min, and stained with 0.1% crystal violet for 30 min.

### γ-H2AX immunofluorescence assay

γ-H2AX is a cellular DNA damage marker; γ-H2AX expression increases when the cells undergo breaks in the double-stranded DNA structure (Singh, Ozturk et al. 2015). Following the instructions of the DNA damage kit, the cells were fixed using 4% paraformaldehyde. γ-H2AX rabbit monoclonal antibody was added, and the cells were incubated at room temperature for 1 h. Subsequently, an anti-rabbit 488 antibody was added and incubated at room temperature for 1 h. Finally, 4’,6-diamidino-2-phenylindole was added and a fluorescence microscope was used to observe DNA damage in different groups of cells.

### Apoptosis immunofluorescence assay

To analyze apoptosis under a microscope, the Annexin V-AbFluor™ 488/PI Double Staining Apoptosis Detection Kit was operated following the User’s Guide for the Annexin V-AbFluor™ 488/PI double staining apoptosis detection Kit. The processed cells were mixed with staining reagents and incubated for 30 min away from light. Subsequently, apoptosis was observed under a fluorescence microscope (Canon; Japan).

### Statistical analysis

Error bars in the experimental results are presented as mean ± standard deviation (mean ± SD) or median ± 95% confidence interval (median ± 95% CI). Each statistical data point reflects the mean of a minimum of three parallel experiments. The t-test was applied to examine differences in ZNF281 expression. Cox regression was employed to assess the correlation between ZNF281 and GC prognosis. The bioinformatics analysis was conducted using R v 4.0.1. Results were analyzed using GraphPad Prism 9 and ImageJ, and experiments were replicated three times. The p-values were calculated with a two-tailed test, with *p* < 0.05 indicating statistical significance.

## Results

### Differential expression and survival analysis

**Fig. 1:**
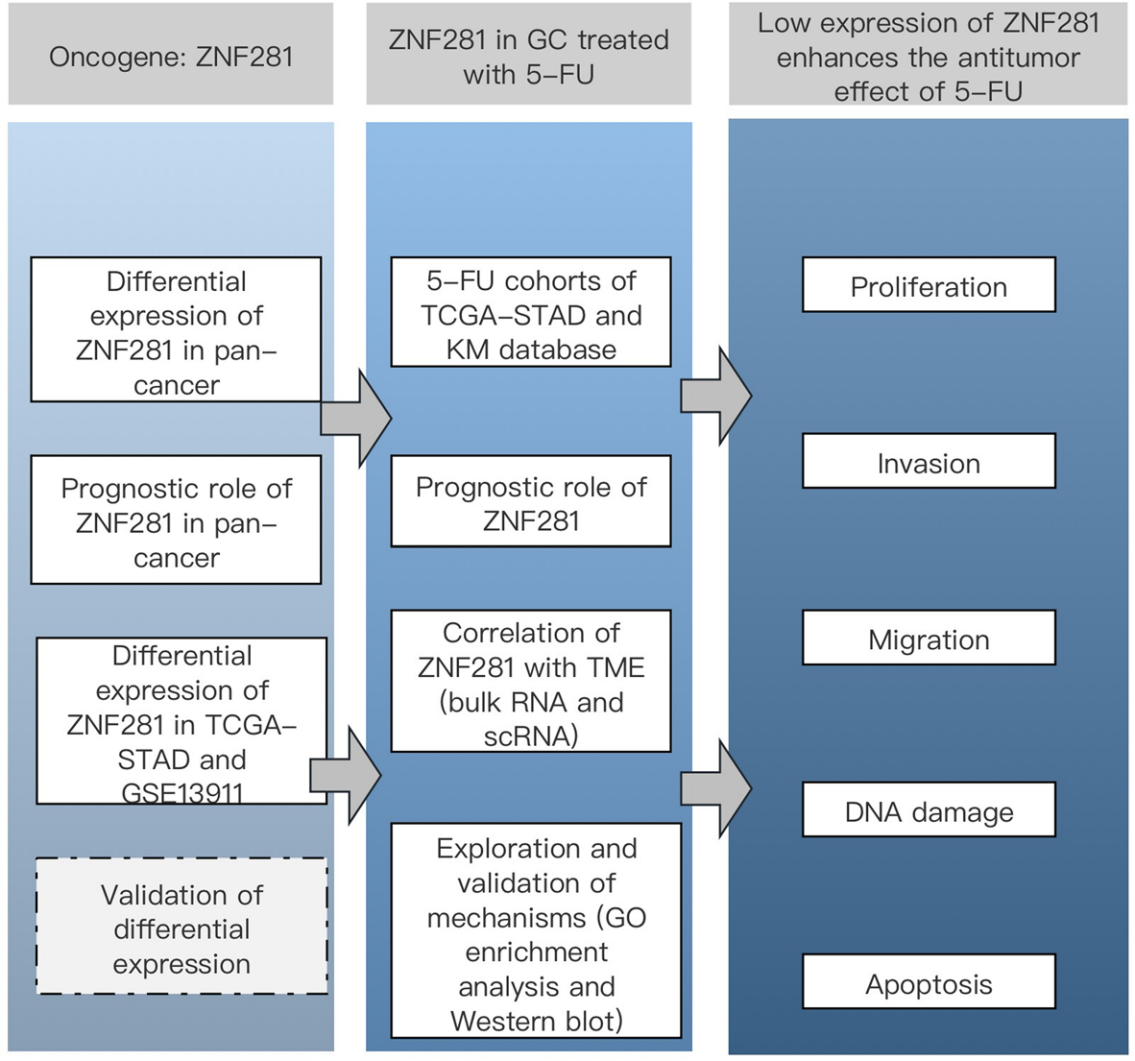
Flow chart of this study

**Fig. 2:**
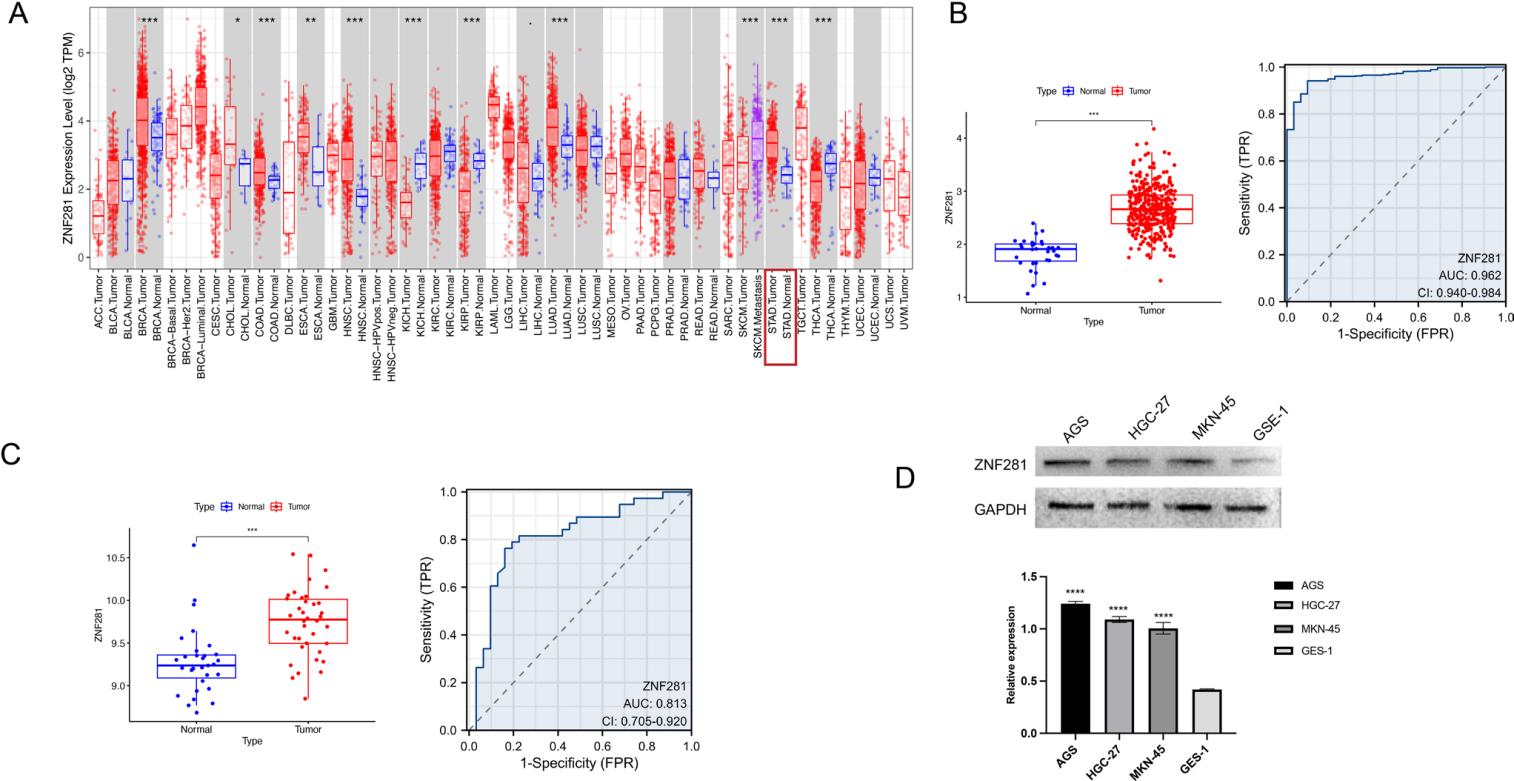
Analysis and validation of the differential expression of ZNF281: (**A**) Differential expression of ZNF281 in pan-cancer. (**B**) Differential expression (left) and ROC curve (right) of ZNF281 in the TCGA-STAD cohort. (**C**) Differential expression(left) and ROC curve(right) of ZNF281 in the GSE13911 cohort. (D). Differential expression of ZNF281 in GC cells and normal cell

All steps of this study were integrated in Fig. 1. ZNF281 highly expressed in BRCA (Breast invasive carcinoma), COAD (Colon adenocarcinoma), CHOL (Cholangiocarcinoma), ESCA (Esophageal carcinoma), HNSC (head and neck squamous cell carcinoma), LUAD (lung adenocarcinoma) and STAD (Stomach adenocarcinoma) (Fig. 2A). In the TCGA-STAD cohort, ZNF281 expression was higher in GC than in normal tissues. The ROC curve illustrated that ZNF281 displayed good diagnostic value in GC (area under the ROC, AUC = 0.962: confidence interval, CI = 0.940–0.984) (Fig. 2B). The GSE13911 database results suggested high ZNF281 expression in GC samples. The AUC illustrated that ZNF281 displayed good diagnostic value (AUC = 0.962, CI = 0.940–0.984) (Fig. 2C). The expression of ZNF281 was higher in GC cells than in normal epithelial cells (Fig. 2D).

### ZNF281 is an independent prognostic factor

**Fig. 3:**
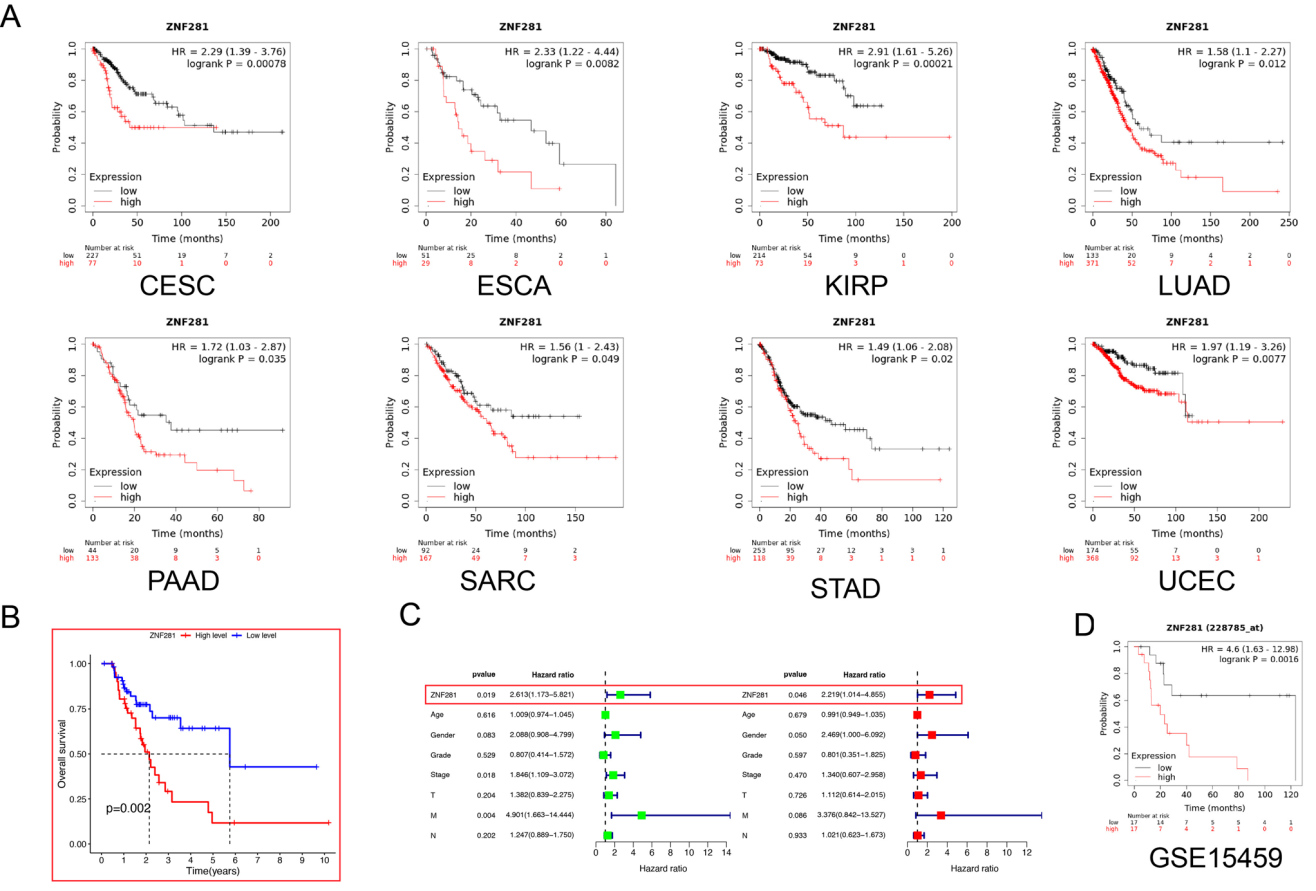
Relationship between expression of ZNF281 and prognosis (**A**) ZNF281 expression correlated with survival in eight cancers. (**B**) In the 5-FU cohort, patients with low ZNF281 expression showed a better prognosis. (**C**) Univariate and multivariate Cox regression analyses identified ZNF281 as an independent influence on prognosis in patients treated with 5-FU chemotherapy. (**D**) Low expression of ZNF281 associated with better prognosis in GSE15459 validation data

Pan-cancer survival analysis showed that ZNF281 expression was strongly associated with the prognosis of CESE (Cervical squamous cell), ESCA, KIRP (Kidney renal papillary cell carcinoma), LUAD, PAAD (Pancreatic adenocarcinoma), SARC (Sarcoma), STAD and UCEC (Uterine Corpus Endometrial Carcinoma) (Fig. 3A). A better prognosis was observed in patients with low ZNF281 expression in the 5-FU cohort (Fig. 3B). Results of univariate and multivariate Cox regression analyses suggested that ZNF281 can be used as an independent risk factor for 5-FU cohort (univariate Cox: HR = 2.163, *P* = 0.019; multivariate Cox: HR = 2.219, *P* = 0.046) (Fig. 3C). In the GC chemotherapy cohort of the KM database, patients with low ZNF281 expression had a better prognosis (Fig. 3D).

### Relationship between ZNF281 expression and TME

**Fig. 4:**
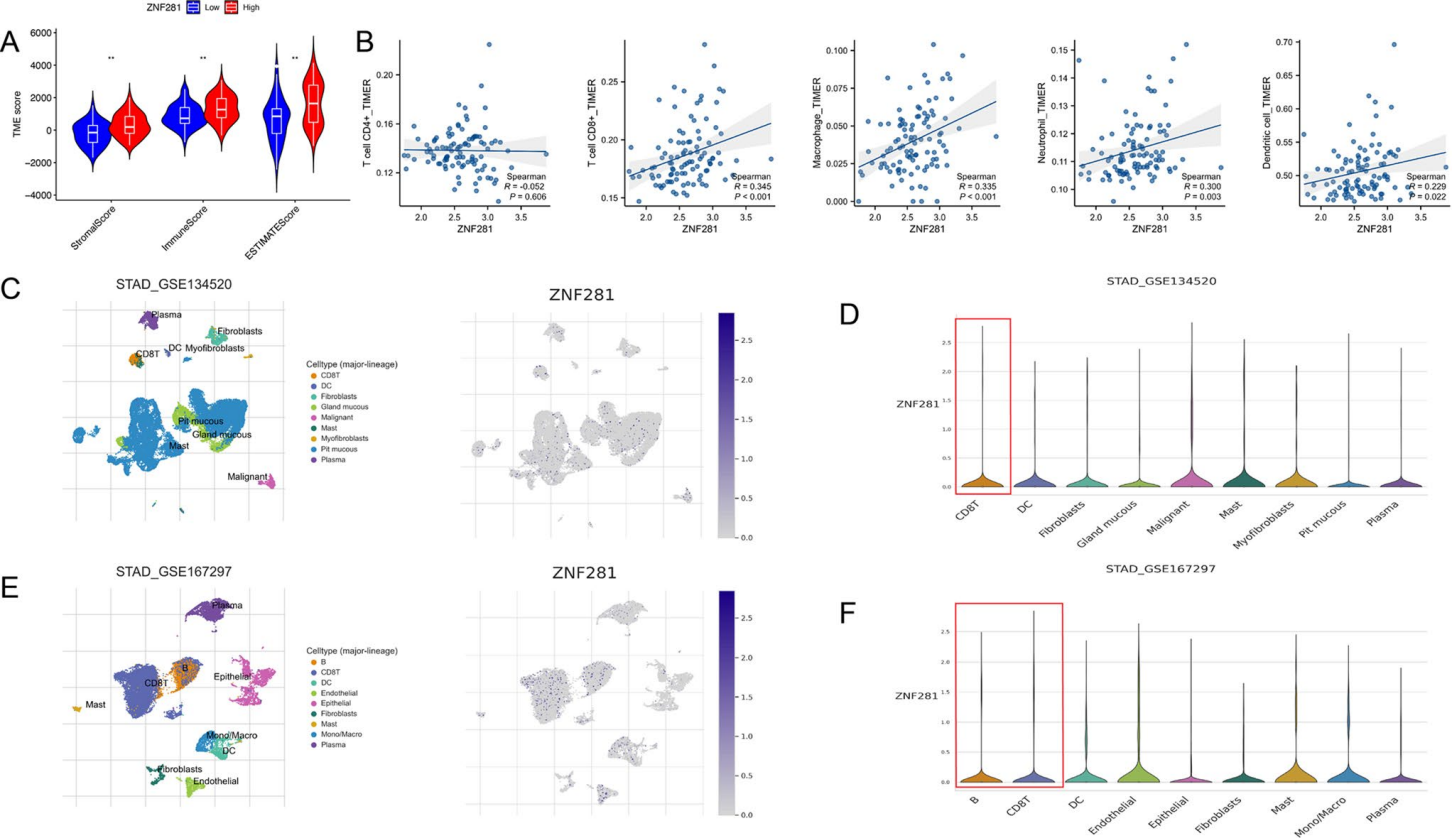
Relationship between ZNF281 and TME (**A**) TME score (5-FU cohort) differences between high and low ZNF281 expression groups. (**B**) Correlation between ZNF281 expression and immune infiltration score (5-FU cohort). (**C**) The GSE134520 dataset was annotated into nine cell clusters (left), and the distribution of ZNF281 in each cluster. (**D**) ZNF281 is highly expressed in CD8T cells. (**E**) The GSE167297 dataset was annotated into nine cell clusters (left), and the distribution of ZNF281 in each cluster. (**F**) ZNF281 is highly expressed in CD8T cells

Three scores of TME were higher in the high ZNF281 group than in the low (Fig. 4A). The results of immune infiltration showed a higher correlation between ZNF281 expression and the abundance of CD8T cells and macrophages (CD8T cell: *R* = 0.345, *P* < 0.001; macrophages: *R* = 0.335, *P* < 0.001) (Fig. 4B). The scRNA analysis verified the results of immune infiltration, and in GSE134520 and GSE167297, ZNF281 expression was upregulated in CD8T lymphocytes (Fig. 4C-F).

### Relationship between ZNF281 and Wnt/β-catenin pathway

**Fig. 5:**
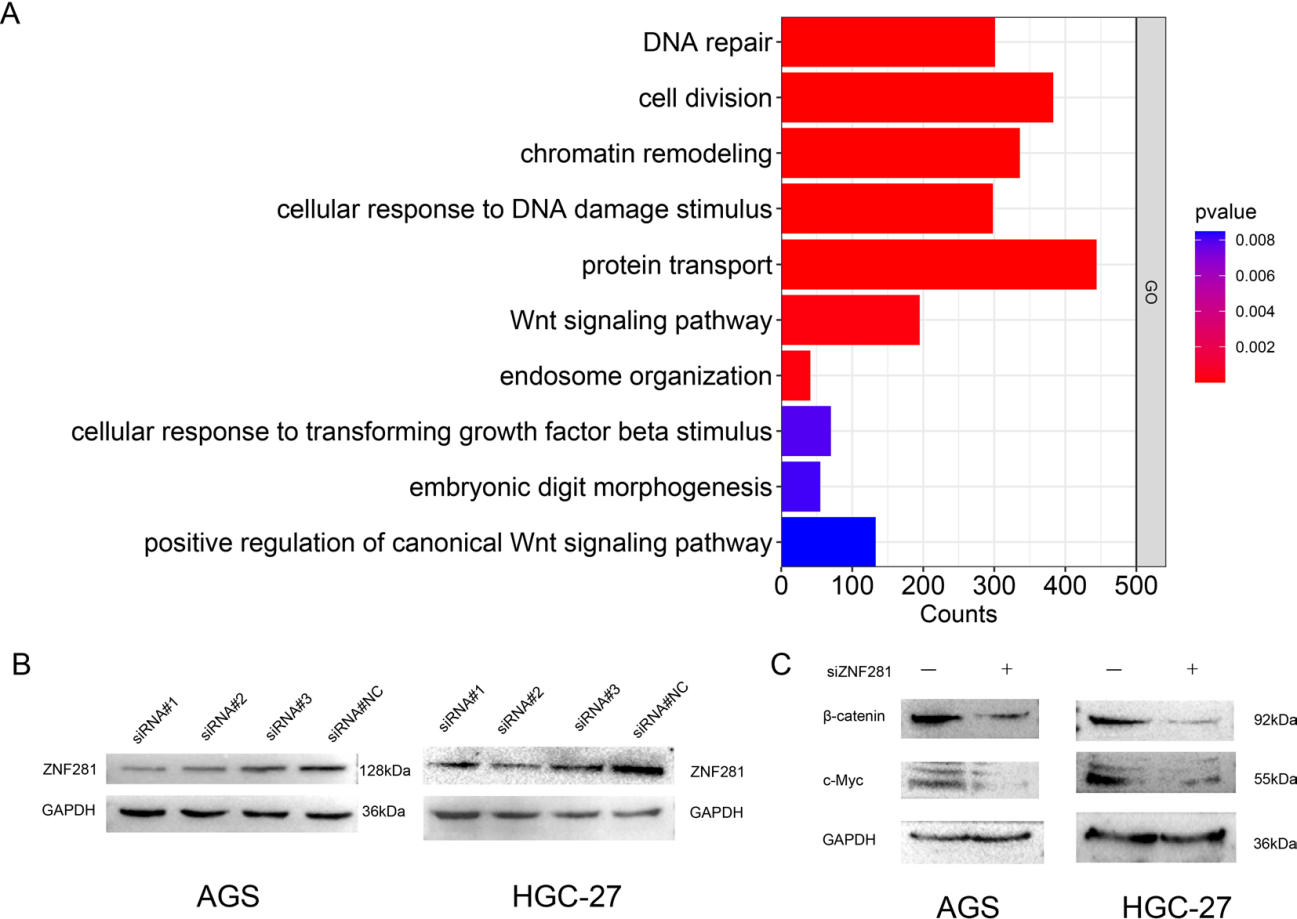
Identification and validation of the relationship between ZNF281 and the Wnt/β-catenin pathway (**A**) GO enrichment analysis of genes positively associated with the expression of ZNF281. (**B**) Selection of highest efficiency siRNAs for AGS (left) and HGC-27 cell (right). (**C**) Knockdown of ZNF281 expression inhibits c-Myc and β-catenin protein expression in AGS (left) and HGC-27 cells (right)

GO enrichment analyses supported the following pathways: “DNA repair” and “Wnt pathway” (Fig. 5A). The siRNA#1 and siRNA#2 transfection demonstrated the highest efficiency in AGS and HGC-27 (Fig. 5B). The expression levels of cMYC and β-catenin were significantly decreased in the siRNA group (Fig. 5C). Therefore, our study identifies Wnt/β-catenin pathway as a potential mechanism of the role of ZNF281 in GC 5-FU treated.

### Cellular toxicity and viability assays

**Fig. 6:**
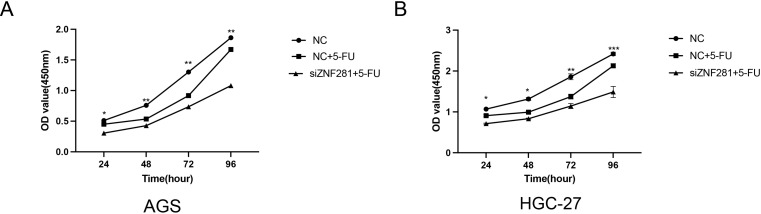
Cell proliferation assay (**A**)Knockdown of ZNF281 enhances the inhibition of AGS cell proliferation by 5-FU. (**B**) Knockdown of ZNF281 enhances the inhibition of HGC-27 cell proliferation by 5-FU

The 5-FU IC50 concentration values of 12.6373 µg/ml and 11.008 µg/ml for AGS and HGC-27, respectively (Fig. 6A). The 5-FU concentration of subsequent experiments was based on the IC50 value at 24, 48, 72, and 96 h. Low ZNF281 expression attenuated the proliferative capacity of 5-FU-treated cells (Fig. 6B).

### Cell migration assays

**Fig. 7:**
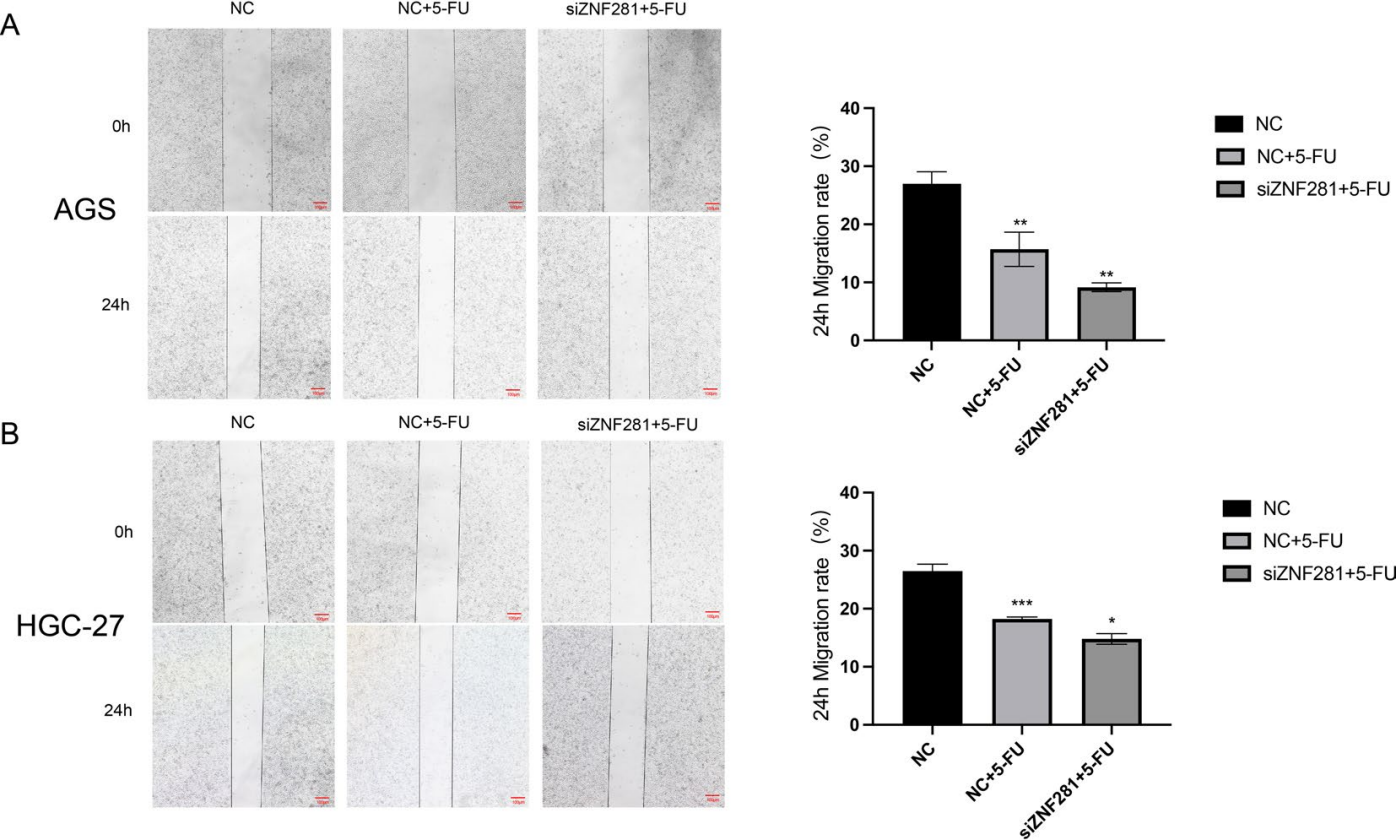
Cell migration assay (**A**)Knockdown of ZNF281 enhances the inhibition of AGS cell migration by 5-FU. (**B**) Knockdown of ZNF281 enhances the inhibition of HGC-27 cell migration by 5-FU

Results of the scratch assay suggested that low ZNF281 expression attenuated the migration ability of 5-FU-treated cells (Fig. 7).

### Cell invasion assay

**Fig. 8:**
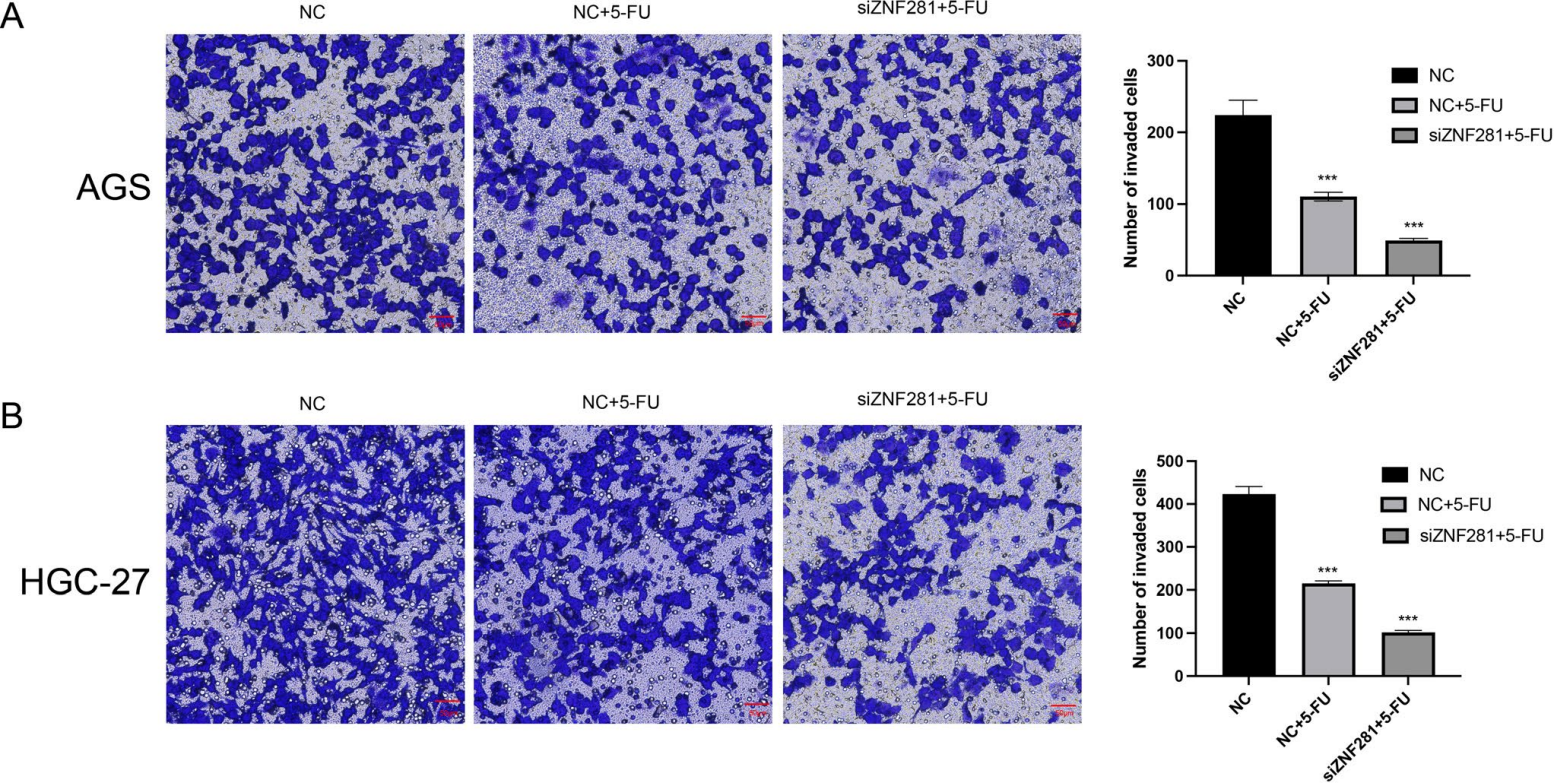
Cell invasion assay (**A**) Knockdown of ZNF281 enhances the invasion of AGS cell migration by 5-FU. (**B**) Knockdown of ZNF281 enhances the inhibition of HGC-27 cell invasion by 5-FU

Results of the Transwell assay demonstrated that low ZNF281 expression attenuated the invasion ability of 5-FU-treated cells (Fig. 8).

### Apoptosis assay

**Fig. 9:**
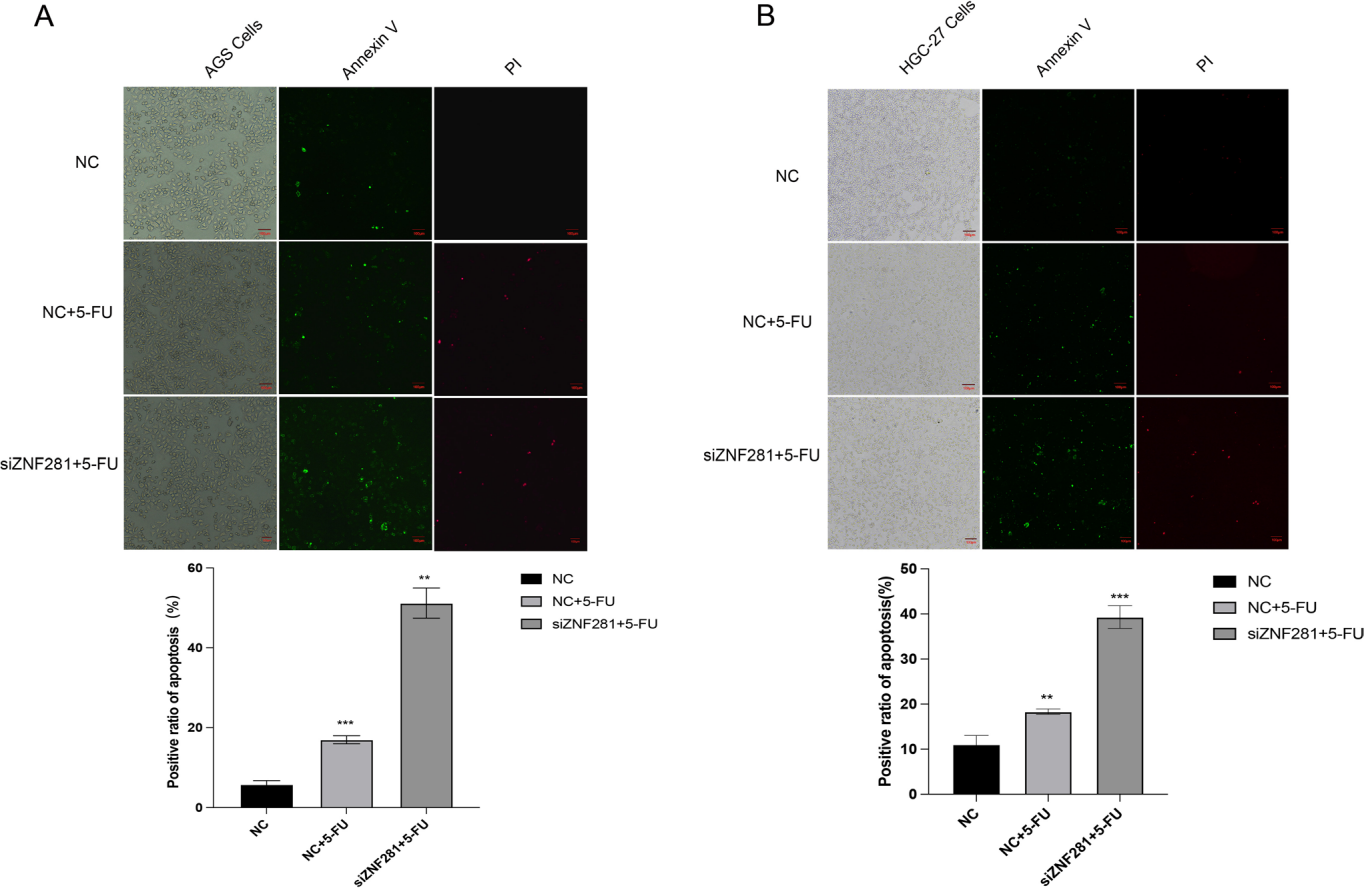
Apoptosis immunofluorescence assay(**A**) Knockdown of ZNF281 enhances 5-FU-induced apoptosis in AGS cells. (**B**) Knockdown of ZNF281 enhances 5-FU-induced apoptosis in HGC-27 cells

In apoptosis assay, pictures without fluorescence are cells, green fluorescence (Annexin v) is for cells in early apoptosis, and the red fluorescence (PI) cells are cells in late apoptosis. The result demonstrated significantly higher apoptosis in the cells treated with 5-FU, compared with the cells not treated with 5-FU. Low ZNF281 expression enhanced the pro-apoptotic efficiency of 5-FU (Fig. 9).

### γ-H2AX immunofluorescence assay

**Fig. 10:**
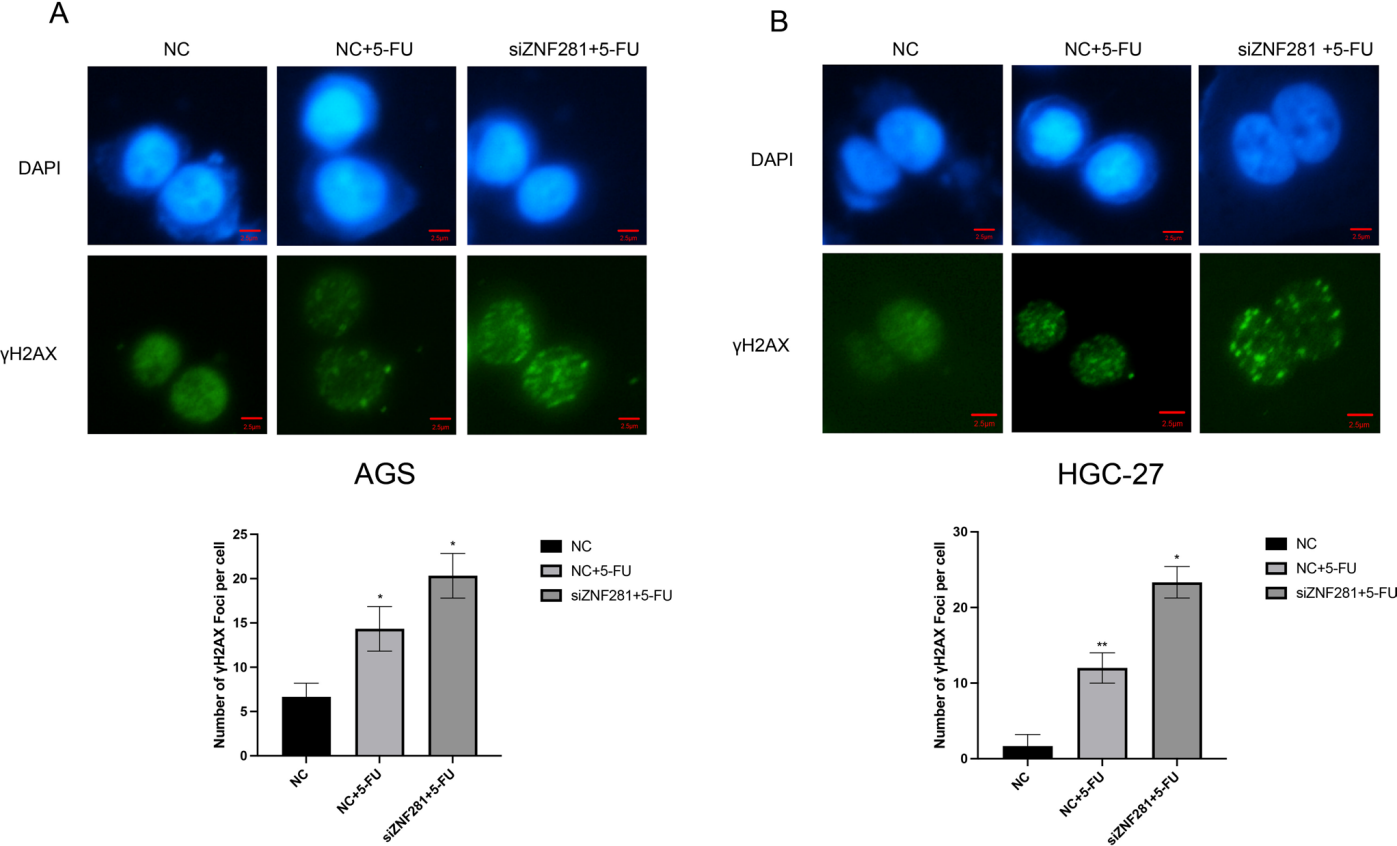
γH2AX immunofluorescence assay (**A**) Knockdown of ZNF281 enhances 5-FU-induced DNA damage in AGS cells. (**B**) Knockdown of ZNF281 enhances 5-FU-induced DNA damage in HGC-27 cells

In γ-H2AX immunofluorescence assay, cell nuclei are stained with blue fluorescence (DAPI), and cells demonstrating DNA damage, indicated by γH2AX positivity, are marked with green fluorescence. The result indicated that the AGS cells that did not receive 5-FU treatment displayed less DNA damage. The percentage of cells that underwent DNA damage increased in the 5-FU treatment group. However, the percentage was highest in the siRNA group (Fig. 10).

## Discussion

Multiple surgeries and chemotherapies have improved the prognosis for several patients with GC. Nonetheless, patients with 5-FU resistance demonstrate poor prognosis. ZNF281 is a potential therapeutic target in multiple types of tumors. However, researchers have not explored its role in GC. Abnormal ZNF281 expression regulates the Wnt/β-catenin pathway affecting tumorigenesis and progression (Qian et al. 2017; Zeng, Hou et al. 2022; Deng, Peng et al. 2023). 5-FU mediates DNA damage and inhibits DNA damage repair in GC cells through the Wnt pathway, thereby promoting apoptosis and inhibiting proliferation, invasion, and migration (Meng et al. 2023). Abnormal activation of the Wnt/β-catenin pathway not only leads to tumor pathogenesis but also induces excessive DNA damage repair in tumor cells, substantially increasing the tolerance of tumor cells to DNA-damaging drugs (Meng et al. 2023). Excessive activation of the Wnt/β-catenin pathway has been associated with cisplatin tolerance in cervical cancer (Chi, Hou et al. 2023). In nephroblastoma, Wnt/β-catenin pathway hyperactivation was associated with carboplatin tolerance (Xie et al. 2021).

We analyzed the expression and role of ZNF281 in pan-cancer. ZNF281 was highly expressed in BRCA, COAD, CHOL, ESCA, HNSC, LUAD and STAD.The expression was associated with prognosis in CESC, ESCA, KIRP, LUAD, PAAD, SARC, STAD and UCEC. Patients with the low-ZNF281 in the 5-FU cohort had a better prognosis, implying that low- ZNF281 may enhance the effects of 5-FU in GC. Cox regression revraled aberrant ZNF281 expression as a prognostic factor independent of other clinical features of 5-FU cohort. TME is an important factor affecting chemotherapy sensitivity and targeting TME may be an important strategy to overcome chemotherapy tolerance. (Zhu, Tian et al. 2021, Ostrowska-Lesko et al. 2024). Many studies have shown that tumour immune cell infiltration affects the efficacy of chemotherapy, radiotherapy or immunotherapy and the prognosis of cancer patients(Batista, Rodvold et al. 2020, Zhang and Zhang 2020). TIMER and scRNA showed that ZNF281 was associated with immune infiltration. We screened the genes positively associated with ZNF281 expression in the TCGA-STAD cohort. Genes of ZNF281 with spearman > 0.3 and p < 0.05 in 5-FU cohort were selected, followed by an enrichment analysis. GO enrichment analyses result was enriched in “DNA repair” and “Wnt pathway”, which suggested the possible effect of ZNF281 on the sensitivity to commonly used DNA damage drugs (5-FU) in GC. Although the DNA damage repair and WNT pathway did not have the most counts in this result, they were statistically significant. So ZNF281 is associated with DNA damage and Wnt/β-catenin pathway and may affect chemotherapy sensitivity in gastric cancer. The pathways with higher enrichment counts were not relevant to the core argument of the present study, but this may indicate that ZNF281 has other biological functions in gastric cancer. This will be further explored in future studies.

In vitro experiments were conducted to validate our hypotheses. Initially, we examined the variations in ZNF281 expression among gastric epithelial GES-1 GC cells (AGS, HGC, and MKN-45). ZNF281 expression was elevated in all GC cells compared to GES-1 cells, with AGS and HGC-27 exhibiting the higher expression. Findings from the CCK-8 assay indicated that 5-FU treatment reduced the proliferative capacity of AGS cells. However, diminished ZNF281 expression in AGS cells further attenuated proliferation following 5-FU treatment. Results of the invasion assay suggested that low ZNF281 expression significantly inhibited the migration ability of AGS after 5-FU treatment. Results of the invasion assay demonstrated that 5-FU inhibited the invasion ability of AGS cells and its effect was amplified by low ZNF281 expression. Migration assays also confirmed that low expression of ZNF281 enhanced the migration inhibition of 5-FU.

To further explore the role of low expression of ZNF281 in enhancing the sensitivity to chemotherapy, we performed apoptosis experiments using the Annexin V/propidium iodide method. Apoptosis was not absent in the control group, which may be attributed to the initiation of the apoptosis program in a few cells. Nonetheless, the 5-FU application increased the percentage of apoptosis. Simultaneous low ZNF281 expression and 5-FU application to NC group significantly increased the apoptotic cells. 5-FU primarily induced DNA damage. To confirm the role of ZNF281, γ-H2AX immunofluorescence was utilized. This step helped us detect DNA damage in the experimental groups. Similar to previous results, the induction of DNA damage by 5-FU was strongest at low ZNF281 expression (Widjaja, Werner et al. 2023).

Bioinformatics analysis and experiments facilitated the identification of low expression of ZNF281 as a novel marker for sensitization to GC chemotherapy. It has been shown that overactivation of the Wnt pathway is one of the mechanisms of tumor drug resistance. Therefore, the inhibition of Wnt is considered to be a sensitizing factor for tumor chemotherapy(Qian et al. 2017; Xiang et al. 2022). Western blot analysis showed a decrease in c-Myc and β-catenin expression after down regulation of ZNF281. This result showed that low expression of ZNF281 may inhibit the Wnt/β-catenin pathway.

In conclusion, to the best of our knowledge, this is the first study to identify ZNF281 as a novel biomarker associated with the prognosis of GC chemotherapy. ZNF281 affects the sensitivity to 5-FU therapy in GC through the Wnt/β-catenin pathway. Low ZNF281 expression may be a potential target for chemotherapy sensitization in GC.

## Electronic supplementary material

Below is the link to the electronic supplementary material.

Supplementary Material 1

Supplementary Material 2

## Data Availability

No datasets were generated or analysed during the current study.

## Abbreviations

GC: Gastric cancer
TCGA: The Cancer Genome Atlas Program database
ZNF281: zinc finger protein 281
5-FU: 5-fluorouracil
STAD: Stomach adenocarcinoma
K–M: Kaplan–Meier
CCK-8: Cell counting Kit-8
BCA: Bicinchoninic acid
GO: Gene Ontology
TIMER: Tumor Immune Estimation Resource
